# Shorter Total Length of Stay After Intraperitoneal Fosfomycin, Metronidazole, and Molgramostim for Complicated Appendicitis: A Pivotal Quasi-Randomized Controlled Trial

**DOI:** 10.3389/fsurg.2020.00025

**Published:** 2020-05-05

**Authors:** Siv Fonnes, Søren Roepstorff, Barbara Juliane Holzknecht, Christoffer Skov Olesen, Joachim Hjalde Halmsted Olsen, Line Schmidt, Rasmus Alder, Sara Gamborg, Tilde Rasmussen, Magnus Arpi, Lars Nannestad Jørgensen, Jacob Rosenberg

**Affiliations:** ^1^Department of Surgery, Centre for Perioperative Optimisation, Herlev and Gentofte Hospital, University of Copenhagen, Herlev, Denmark; ^2^Digestive Disease Centre, Bispebjerg Hospital, University of Copenhagen, Copenhagen, Denmark; ^3^Department of Clinical Microbiology, Herlev and Gentofte Hospital, University of Copenhagen, Herlev, Denmark

**Keywords:** appendicitis, clinical trial, intraabdominal infection, perforation, prophylactic antibiotics

## Abstract

**Background:** We aimed to investigate the difference in the total length of hospital stay (LOS) after intraperitoneal vs. intravenous antibiotic treatment in patients with complicated appendicitis.

**Methods:** We conducted a quasi-randomized prospective clinical trial. The intervention group received 4 g fosfomycin, 1 g metronidazole, and 50 μg recombinant human granulocyte-macrophage colony-stimulating factor intraperitoneally, which was left in the abdominal cavity, immediately after laparoscopic appendectomy. Postoperatively, this group received antibiotics orally. The control group received intravenous antibiotics both during surgery and postoperatively. We primarily evaluated total LOS within 30 days. Furthermore, we evaluated harms and adverse events, Gastrointestinal Quality of Life Index, postoperative complications, and convalescence. Participants were followed for 30 days postoperatively.

**Results:** A total of 12 participants concluded the trial. The total LOS was significantly shorter in the intervention group (six participants, median 13 h; range 2–21 h) than in the control group (six participants, median 84 h; range 67–169 h), *p* = 0.017. Comparable harms and Gastrointestinal Quality of Life Index scores were found in the two groups. The time to return to normal activities was median 6 and 10 days for the intervention and the control group, respectively. There were no serious adverse events related to the trial nor any complications in the intervention group. In the control group, two patients developed intraabdominal abscesses.

**Conclusions:** The intervention group had a significantly shorter total LOS. The study was not powered to assess differences in complications, but the results indicate that the intervention seems to be a safe regimen, which can be investigated further to treat patients with complicated appendicitis.

**Identifiers:** EudraCT no. 2017-004753-16.

**ClinicalTrials:** https://clinicaltrials.gov/ct2/show/NCT03435900?term=NCT03435900&draw=2&rank=1">draw=2&rank=1.

## Introduction

In secondary peritonitis, the abdominal cavity is contaminated with aerobic and anaerobic bacteria from the gastrointestinal tract (1, 2), e.g. *Enterobacterales, Enterococcus faecalis*, and *Bacteroides species* (3). Secondary peritonitis is treated by source control, typically surgery, and empirical antimicrobial therapy (2). A possible regimen could be fosfomycin and metronidazole, which have been shown to cover the relevant aerobic Gram-positive and Gram-negative and anaerobic bacteria, both in previous clinical trials of abdominal surgery (4, 5) and *in vitro* (6). Furthermore, administration of recombinant human granulocyte-macrophage colony-stimulating factor (rhGM-CSF) could improve the local immune response in secondary peritonitis as found in patients undergoing peritoneal dialysis (7) and patients suffering from advanced intraperitoneal malignancies (8).

The infection originates intraperitoneally in secondary peritonitis, but bacteria may spread to the bloodstream (1). Intraperitoneal administration of antimicrobial agents in these patients provides high concentrations at the site of the infection as well as therapeutic plasma concentrations (9). Intraperitoneal administration of antimicrobial agents could, therefore, result in faster and more effective clearance of the local as well as the systemic infection than the standard treatment with intravenously administrated antibiotics. Hence, a shorter antimicrobial regimen or an earlier discharge with an oral regimen after intraperitoneal administration could provide a possible treatment option. This would result in a shorter length of hospital stay (LOS) and a decrease in hospital costs. However, a shorter LOS has no value if the risk of readmission and postoperative complications are increased with the intervention compared with standard treatment.

We aimed to investigate if total LOS could be reduced for patients with complicated appendicitis when treated with intraoperative intraperitoneal administration of fosfomycin, metronidazole and rhGM-CSF followed by an oral antibiotic regimen compared with a standard intravenous antibiotic regimen.

## Materials and Methods

### Trial Design and Approvals

This was a quasi-randomized prospective clinical trial (EudraCT 2017-004753-16). The trial protocol was approved prior to initiation by the Danish Medicines Health Authority (2017113663), the local Ethics Committee (H-17037698), and the Danish Data Protection Agency (HGH-2017-124). The trial was registered at clinicaltrials.gov (NCT03435900) and monitored by the Good Clinical Practice Unit, Copenhagen University Hospital. It is reported according to the CONSORT statement (10) and its extensions regarding harms (11), cluster randomization (12), and feasibility (13).

### Participants and Interventions

The inclusion criteria were: age≥18 years, laparoscopic appendectomy for perforated appendix, and written informed consent. If the participant was a fertile woman a negative urine pregnancy test was required. A perforated appendix was defined as an appendectomy during which the operating surgeon or the supervisor determined the need for postoperative intravenous antibiotic treatment. This usually included a visible appendix perforation, free intra-abdominal pus, visible feces, and/or an abscess.

Exclusion criteria were: inability to understand, read or speak Danish; previous allergic reaction to fosfomycin, metronidazole, rhGM-CSF, or penicillins; diagnostic laparoscopy revealing a normal appendix not requiring an appendectomy or appendicitis without a perforated appendix; other intra-abdominal pathology requiring surgical intervention (diagnosed either during surgery or at a preoperative CT-scan); renal, hepatic, hematological disease; American Society of Anesthesiologists (ASA) physical status >3 (14); body weight >110 kg; surgery converted to open appendectomy; or anticipated compliance problems.

A laparoscopic appendectomy was performed according to routine clinical practice. The intervention group was treated as follows: after appendix removal, a minimum of 500 ml of saline was used for irrigation of the abdominal cavity, and the trial drugs were administered intraperitoneally through the irrigation/suction device and left there. The combination of the trial drugs consisted of a volume of 500.2 ml containing: 4 g fosfomycin (Infectofos, Infectopharm, Germany) diluted in 300 ml of sterile water for injections (Sterilt Vand “SAD,” Amgros I/S, Denmark), 1 g metronidazole (Metronidazol “B. Braun,” B. Braun, Germany) corresponding to a volume of 200 ml and 50 μg molgramostim, which is rhGM-CSF expressed in *Escherichia coli* (Repomol, Reponex Pharmaceuticals aps., Denmark), which is purified prior to clinical use, corresponding to a volume of 0.2 ml. All the drugs were combined prior to instillation but administered immediately after mixing to ensure full antimicrobial effect (6). Postoperatively, participants received orally administered antibiotics: 500 mg amoxicillin/125 mg clavulanic acid and 500 mg metronidazole administered three times daily for 3 days.

The control group received standard intravenous antibiotic agents during surgery (either 1.5 g cefuroxime or 4 g piperacillin/500 mg tazobactam and 1 g metronidazole) and a minimum of 500 ml saline was used for irrigation of the abdominal cavity. Postoperatively, participants received 3 days of intravenously administered antibiotic agents: 4 g piperacillin/500 mg tazobactam and 500 mg metronidazole. These doses were administered three times daily for a minimum of 3 days.

All study participants could receive preoperative intravenously administered antibiotic agents; however, for the intervention group, intravenous administration immediately before initiation of surgery was avoided.

All participants could be discharged when the Postanesthesia Recovery Score for Ambulatory Patients (PARSAP score) (15) was ≥18. Participants in the control group were first discharged after finishing their intravenous antibiotic treatment.

### Outcomes

The primary outcome was total LOS in the two groups, defined as the number of hours in hospital after the end of the surgery and until 30-day follow-up including any readmissions related to the laparoscopic appendectomy or appendicitis. The secondary outcomes included the following:

#### Questionnaires

The questionnaire Gastrointestinal Quality of Life Index (GIQLI) (16) was filled in by participants 10 days (±2 days) and 30 days (±3 days) postoperatively. GIQLI has previously been translated from English to Danish (17). A questionnaire of the participant's subjective harms was filled in by the participants at the first postoperative day and 10 days (±2 days) postoperatively. Both questionnaires in Danish were face validated in patients with appendicitis prior to initiation of the trial.

#### Complications

Postoperative complications were graded according to the Clavien-Dindo classification (18). Furthermore, specific information regarding deep surgical site infections requiring surgical drainage, intraabdominal abscesses requiring drainage, readmissions, and reoperations was collected. The information was collected during the hospital stay after the surgery, 10 days postoperatively (±2 days) when the patients had their sutures removed by the trial personnel, and 30 days (±3 days) postoperatively by review of the patients' medical records and a planned telephone interview.

#### Convalescence

Participants were asked both when they could return to normal activities and how long the period of sick leave (absence from work) had lasted. Data were collected up to 30 days (±3 days) postoperatively.

#### Adverse Events

These were registered by trial personnel during admission, 10 days (±2 days) postoperatively when the patients had their sutures removed by the trial personnel and 30 days (±3 days) postoperatively through review of the patient's medical records and at a planned telephone interview. Adverse events were defined as any untoward medical occurrence including unfavorable and unintended signs (such as abnormal laboratory findings), symptoms, and disease as defined by ICH-GCP (19). Serious adverse events (SAE) or serious adverse drug reactions were defined as any untoward medical occurrence that at any dose resulted in death, was life-threatening, required inpatient hospitalization or prolongation of existing hospitalization, resulted in persistent or significant disability or incapacity, or resulted in a congenital anomaly or birth defect as defined by ICH-GCP (19).

### Sample Size and Randomization

We performed a power calculation for the trial. The cumulative 30-day total LOS was expected to be 72 h with a standard deviation of 12 h in the control group. When alpha was set at 0.05, beta was set at 0.80, and a minimal relevant difference between the groups was set at 48 h, a randomized design required four patients in each group, a total of eight patients. However, as we expected data not to be normally distributed and planned to use a quasi-randomized design, we decided to include six patients in each group, a total of 12 patients, to ensure sufficient power. Participants were included until data on the primary outcome at day 30 were secured for six patients in each group. The intervention group was recruited at one hospital (Herlev Hospital) and the control group at another hospital (Bispebjerg Hospital). The two hospitals are located 9 km apart. Thus, the allocation was not concealed, and participants, care providers, and outcome assessment were not blinded to the intervention.

### Statistical Methods

Data were analyzed using SAS Enterprise Guide 7.1 (SAS Institute Inc., USA). Details on the statistical methods can be seen in the statistical analysis plan in **Supplements**. In short, continuous numerical values were reported as median and range if not normally distributed. Not normally distributed continuous data were analyzed with non-parametric statistics: Mann-Whitney U-test. Binary, categorical data were reported as numbers and proportions in %. We analyzed these with Chi-square-test, and if any of the expected cell counts were <5, then *p*-values from the Fisher's exact test is reported. A *p*-value ≤ 0.05 was considered statistically significant.

## Results

**Figure 1:**
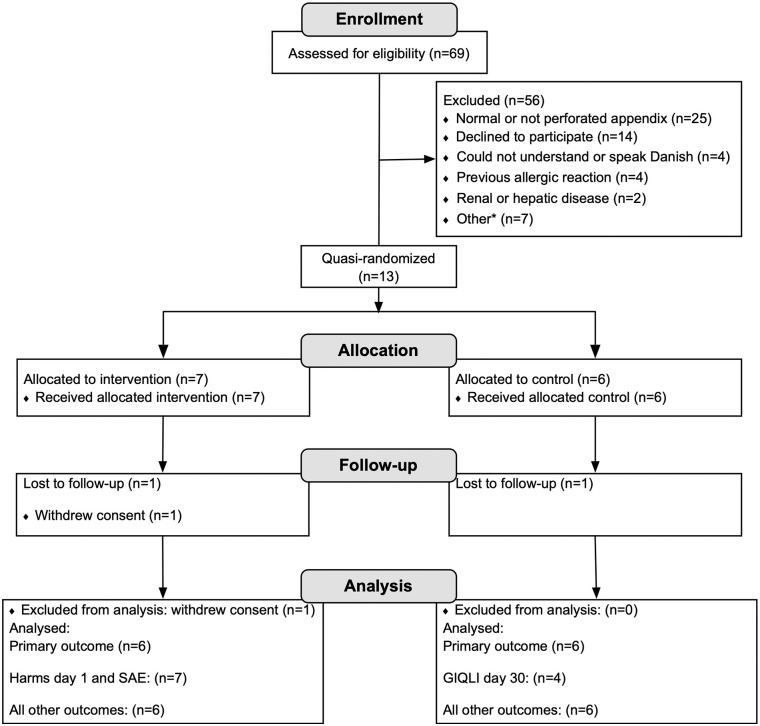
Flowchart of the screened, enrolled, allocated, and analyzed patients in the trial. *Other reasons for exclusion (*n*): breastfeeding (1), hematological disease (1), other intra-abdominal disease requiring surgical intervention (1), administration of intravenous metronidazole prior to surgery (intervention group, 1), other unspecified (2), logistic circumstances (1). SAE, serious adverse event.

Eligible patients were recruited at the Department of Surgery, Herlev Hospital (intervention group) and the Digestive Disease Centre, Bispebjerg Hospital (control group) from 14th February 2018 to 17th June 2018. A total of 13 participants were included, as one patient withdrew consent 3 days after surgery. Therefore, data on the primary outcome could not be retrieved and another patient was included according to the predefined protocol. The screening process is depicted in Figure 1. Last day of follow-up was 17th July 2018, where the study ended. Six participants in the intervention and in the control group completed full follow-up. There were missing data on one outcome in the control group and data from the participant who withdrew consent until the third postoperative day. The exact number of analyzed participants for outcomes are, therefore, presented in Figure 1. The demographics and baseline characteristics of all included patients are presented in Table 1.

**Table 1:** Demographics and baseline characteristics of the 13 included patients for continuous variables in median [range] and for categorical variables in numbers (per cent).

**Admission**		**Intervention group (*n* = 7)**	**Control group (*n* = 6)**	***p*-value**
Age, years		52 [21–73]	28 [18–55]	0.13^†^
Sex, female		4 (57%)	1 (17%)	0.27^‡^
Height, cm		173 [163–185]	185 [168–187]	0.06^†^
Weight, kg		82 [65–105]	92 [73–107]	0.78^†^
Body Mass Index, kg/m^2^		29 [24–34]	28 [21–33]	0.63^†^
ASA score	I: II:	4 (57%) 3 (43%)	6 (100%) 0 (0%)	0.19^‡^
Preoperative antibiotics^*^				
Times administered		2 [0–8]	2 [0–4]	0.54^†^
**Surgery**				
Length, hours:minutes		01:22 [01:03–02:33]	01:20 [00:38–02:37]	0.78^†^

One patient withdrew consent on the third postoperative day. ASA, American Society of Anesthesiologists.14

* Included the following antibiotics: ampicillin, cefuroxime, gentamicin, piperacillin/tazobactam, and metronidazole.

† Mann-Whitney U-test,

‡ *Fisher's exact test*.

### Total LOS

The total LOS was significantly lower in the intervention group (median 13 h; range 2–21 h) than in the control group (median 84 h, range 67–169 h), *p*-value = 0.017 (Mann-Whitney U-test).

### Questionnaires

The total GIQLI score seemed to increase from 10th to 30th postoperative day in both groups (Table 2). The score regarding the domain of symptoms seemed to increase in both groups. The scores for the domain of social function seemed to decrease in both groups from 10th to 30th postoperative day.

**Table 2:** The median [range] scores of the Gastrointestinal Quality of Life Index (GIQLI) (16) overall and for each item of the intervention and the control group postoperatively.

	**10th postoperative day**		**30th postoperative day**	
**GIQLI**	**Intervention group (*****n*** **=** **6)**	**Control group (*****n*** **=** **6)**	**Intervention group (*****n*** **=** **6)**	**Control group (*****n*** **=** **4)**
**Total**	112 [102–119]	102 [86–115]	118 [73–126]	120 [114–120]
Symptoms	62 [57–69]	55 [45–66]	68 [43–72]	69 [68–69]
Emotions	12 [8–15]	11 [5–14]	13 [8–14]	14 [12–−15]
Physical function	18 [14–26]	20 [15–23]	21 [9–28]	23 [21–27]
Social function	14 [11–16]	13 [10–14]	9.5 [6–12]	9 [8–12]
Medical treatment	4 [3–4]	3 [2–4]	3.5 [1–4]	4 [3–4]

*The maximal scores of GIQLI are total 144, symptoms 76, physical function 28, emotions 12, social function 14, and medical treatment 4*.

An overview of the reported harms is presented in Table 3. The most commonly reported harms were discomfort when breathing deeply, diarrhea, and bloating. Discomfort when breathing deeply was present for both groups within 24 h after surgery and 10 days postoperatively. Diarrhea was reported only in the interval between 24 h and 10 days postoperatively in both intervention (67%) and control group (67%). Bloating was reported at 24 h and within the first 10 days postoperatively in both groups; however, the prevalence increased over time from 14 to 50% in the intervention group and from 50 to 83% in the control group. Other reported harms within 24 h after surgery were nose bleeding (17%) and dark urine (17%) in the intervention group and pain in the penis during urination (14%) in the control group. Other reported harms within 10 days postoperatively were high pulse (14%) and pain (14%) in the intervention group and vomiting (14%) in the control group.

**Table 3:** The reported harms in the intervention and the control group.

	**Within 24 h**		**10th postoperative day**	
**Organ system**	**Yes, to some degree**, ***n*** **(%)**		**Yes, to some degree**, ***n*** **(%)**	
**Complaint**	**Intervention, 7**	**Control, 6**	**Intervention, 6**	**Control, 6**
**Central nervous system**				
Dizziness	1 (14%)	1 (14%)	1 (17%)	3 (50%)
**Cardio-pulmonary**				
Discomfort when breathing deeply	3 (43%)	3 (43%)	4 (67%)	4 (67%)
Coughing	2 (29%)	2 (29%)	2 (33%)	4 (67%)
**Gastrointestinal**				
Bloating	1 (14%)	3 (50%)	3 (50%)	5 (83%)
Flatulence	2 (29%)	3 (50%)	5 (83%)	6 (100%)
Diarrhea	1 (14%)	0	4 (67%)	4 (67%)
Mild wound secretion	1 (14%)	1 (17%)	0	1 (17%)

“*Yes, to some degree” corresponds to any of the following statements: “a little of the time,” “some of the time,” and “all of the time”*.

### Complications

Of the 12 evaluable participants, there were no complications in the intervention group and two complications in the control group within 30 days after surgery. These complications consisted of two intraabdominal abscesses in two participants (33%). The Clavien-Dindo grade of both complications was 3a, which are complications requiring radiological intervention that are not under general anesthesia. The complications meant that one participant had a prolonged hospital stay and one participant was readmitted.

### Convalescence

In the intervention group, one participant had not returned to normal activity at day 30. Median time to return to normal activities was 6 days [range 1 to >30 days]. In the control group, the median time to return to normal activities was 10 days [range 4–28 days].

One participant in the intervention group was still on sick leave at 30 days postoperatively. The period of sick leave for the intervention group was median 13 days [range 1 to >30 days]. The control group had a median sick leave of 12 days [range 4–28 days].

### Adverse Events

There were no unexpected adverse events in either the intervention or control group. There were six adverse events (0–2 per participant) in the intervention group. These included nausea (17%), diarrhea (67%), and pain in the right lower quadrant (17%). Six adverse events (0–3 per participant) were found in the control group. These included diarrhea (50%), nausea and vomiting (17%), vomiting (17%), and haematoma (17%).

Four SAE were reported: two unrelated and two potentially related to the trial. One unrelated SAE occurred in the intervention group. The admission for appendicitis was prolonged due to influenza, and this participant withdrew consent. There were two potentially related and one unrelated SAE in the control group. The potentially related SAEs included prolonged admission due to an intraabdominal abscess that required both drainage and prolonged antibiotic treatment (*n* = 1) and readmission within 30 days due to an intraabdominal abscess requiring drainage (*n* = 1). The unrelated SAE was readmission within 30 days due to diverticulitis.

## Discussion

The total LOS within 30 days after surgery was significantly shorter in the intervention group than the control group in this quasi-randomized prospective clinical trial. Harms and GIQLI scores were comparable for the two groups. There seemed to be a shorter time to return to normal activities in the intervention group. No SAEs related to the trial were reported in the intervention group. Two possibly related SAEs were registered in the control group, prolonged hospitalization and readmission, both due to the intraabdominal abscess.

Standard treatment for complicated appendicitis includes intravenously administered antibiotics, which requires the patient to stay in hospital. The duration of the postoperative antimicrobial treatment varies between countries and even hospitals. Observational cohort studies have shown that the risk of infectious complications is the same after treatment for 3 and 5 days (20, 21). The median hospital stay, however, was shorter for patients treated for 3 days (21). A postoperative oral antibiotic regimen could lead to a further reduction in the hospital stay and in overall costs (22). We found that it was feasible to discharge participants in the intervention group with a PARSAP score of ≥18 within 24 h postoperatively, except in one case when the participant also suffered from influenza. None of the participants in the intervention group had to be readmitted within 30 days postoperatively. This resulted in a shorter median total LOS for the intervention group of 13 h compared with 86 h in the control group.

Complications are feared after laparoscopic appendectomy for complicated appendicitis. Therefore, prolonged antimicrobial treatment is administered. In the Western world, these complications are rare (22). For instance, 12% of patients will suffer from a Clavien-Dindo grade 3a complication, which is a complication requiring radiologic intervention, and the risk of an intraabdominal abscess is only around 7% (22). In our trial, the two complications were graded as Clavien-Dindo grade 3a and included intraabdominal abscess in two participants in the control group, but our trial was not powered to show a difference in complications between the two groups. A randomized trial assessing complications would require more than 1,000 participants in each group due to the low prevalence of complications, especially the intraabdominal abscesses. However, we find it reassuring that there have been no infectious complications in any of the participants receiving the trial drugs both in the present and the previous trial in a healthier population (23). This is in line with our investigations made prior to the initiation of clinical trials, where we documented the antimicrobial effect of the trial treatment *in vitro* (6). The effect of antimicrobial agents, however, is overall challenged by emerging resistance, which also is a concern for intraabdominal infections (3). Fosfomycin is especially interesting in that context as it has maintained activity against a variety of bacteria with acquired resistance and is used for treatment of serious infections with multidrug-resistant bacteria (24). Resistance against fosfomycin may occur, especially during prolonged use as monotherapy. A meta-analysis found a pooled estimate of 3% for the emergence of fosfomycin resistance during fosfomycin monotherapy (24). However, in a setting where fosfomycin is used as a single dose for a community-acquired infection, it is unlikely that emergence of resistance will be a major problem.

We found that comparable harms were reported both in the intervention and control group. The reported harms were most likely associated with the antibiotics, surgery, and/or anesthesia and not the route of drug administration. The harm that was most often reported was diarrhea, which occurred in 67% of participant both in the intervention and the control group within 30 days postoperatively. Diarrhea is a commonly reported side effect of intravenous fosfomycin (1%) (24), intravenous metronidazole (25), and intravenous piperacillin/clavulanic acid (1–10%) (26). It is, however, reported to be an uncommon side effect of cefuroxime (27). The prevalence of diarrhea in both groups in the present trial was higher than in the summary of product characteristics of the drugs. The reason for the higher prevalence could be a longer period of follow-up and a protocolled assessment of harms.

This quasi-randomized clinical trial has several strengths. We conducted both *in vitro* investigations (6) and a clinical trial in a healthier population (23) prior to the present trial in patients suffering from complicated appendicitis. We assessed the total LOS and not only the participants' immediate postoperative hospital stays. Therefore, any complications, which required readmission, that arose within 30 days postoperatively would affect the median total LOS in both groups and thus reflect a lack of feasibility. We assessed all harms and adverse events thoroughly through evaluation of the participant within 24 h after surgery, a follow-up visit 10 days postoperatively, and by a telephone interview 30 days postoperatively. Furthermore, we ensured a full follow-up for the first 30 days postoperatively of all participants through electronic medical records regarding complications. This study is generalizable as it was conducted in an average population suffering from complicated appendicitis, as we included males and females and there were no age limitations. Some limitations must, however, be mentioned. The sample size was small. Although intraabdominal abscess is one of the most clinically significant outcomes, it is a rare complication, and therefore, many participants would be needed to assess the effect on this outcome. This smaller pilot trial seemed ethically more correct to assess the safety of the intervention and the total LOS. The latter has a high impact on both the participants and the total cost of their treatment, but it remains a limitation that the trial does not have the power to assess a difference or equivalence in complications. Therefore, larger studies assessing complications are needed to confirm the results of our pilot study. Furthermore, the quasi-randomized design meant that no actual randomization took place, thus there is a risk of selection bias, and this study design was chosen for practical reasons. However, the standard treatment of complicated appendicitis, including operation techniques did not differ between the two departments.

In conclusion, this quasi-randomized clinical trial found that the intervention group had a shorter total LOS than the control group, and that harms were comparable. It, therefore, seems to be relevant and safe to investigate this trial treatment further.

## Data Availability Statement

The datasets for this article are not publicly available because of Danish legislation. Requests to access the datasets should be directed to Siv Fonnes, siv.fonnes@gmail.com.

## Ethics Statement

The studies involving human participants were reviewed and approved by the local Ethics Committee of the Capital Region of Denmark (H-17037698).

## Author Contributions

SF, BH, MA, and JR: substantial contributions to the conception or design of the work. SR, CO, JO, LS, RA, SG, TR, and LJ: acquisition. SF, BH, MA, LJ, and JR: analysis or interpretation of data for the work. SF: drafting the work. SR, BH, CO, JO, LS, RA, SG, TR, MA, LJ, and JR: revising it critically for important intellectual content. SF, SR, BH, CO, JO, LS, RA, SG, TR, MA, LJ, and JR: provide approval for publication of the content, agree to be accountable for all aspects of the work in ensuring that questions related to the accuracy, or integrity of any part of the work are appropriately investigated and resolved.

## Conflict of Interest

This study was funded by a grant from Reponex Pharmaceuticals ApS to the Department of Surgery, Herlev Hospital. The company did not have any influence on the study design, study conduct, or writing of the manuscript. The remaining authors declare that the research was conducted in the absence of any commercial or financial relationships that could be construed as a potential conflict of interest.

## References

[1] Ross JT Matthay MA Harris HW. Secondary peritonitis: principles of diagnosis and intervention. BMJ. (2018) 361:k1407. 10.1136/bmj.k1407 29914871 PMC6889898

[2] Pieracci FM Barie PS. Management of severe sepsis of abdominal origin. Scand J Surg. (2007) 96:184–96. 10.1177/145749690709600302 17966743

[3] Sartelli M Catena F Ansaloni L Leppaniemi A Taviloglu K van Goor H. Complicated intra-abdominal infections in Europe: a comprehensive review of the CIAO study. World J Emerg Surg. (2012) 7:36. 10.1186/1749-7922-7-36 23190741 PMC3539964

[4] Andåker L Burman LG Eklund A Graffner H Hansson J Hellberg R. Fosfomycin/metronidazole compared with doxycycline/metronidazole for the prophylaxis of infection after elective colorectal surgery. A randomised double-blind multicentre trial in 517 patients. Eur J Surg. (1992) 158:181–5. 1356459

[5] Andåker L Höjer H Kihlström E Lindhagen J. Stratified duration of prophylactic antimicrobial treatment in emergency abdominal surgery. Metronidazole-fosfomycin vs. metronidazole-gentamicin in 381 patients. Acta Chir Scand. (1987) 153:185–92. 3300120

[6] Fonnes S Holzknecht BJ Gasbjerg LS Weisser JJ Hallberg HW Arpi M. The combination of fosfomycin, metronidazole, and recombinant human granulocyte-macrophage colony-stimulating factor is stable *in vitro* and has maintained antibacterial activity. Drug Res. (2018) 68:349–54. 10.1055/s-0043-123933 29258152

[7] Selgas R Fernández de Castro M Jiménez C Cárcamo C Contreras T Bajo MA. Immunomodulation of peritoneal macrophages by granulocyte-macrophage colony-stimulating factor in humans. Kidney Int. (1996) 50:2070–8. 10.1038/ki.1996.531 8943492

[8] Toner GC Gabrilove JL Gordon M Crown J Jakubowski AA Meisenberg B. Phase I trial of intravenous and intraperitoneal administration of granulocyte-macrophage colony-stimulating factor. J Immunother Emphasis Tumor Immunol. (1994) 15:59–66. 10.1097/00002371-199401000-00008 8110732

[9] Fonnes S Weisser JJ Holzknecht BJ Arpi M Rosenberg J. The plasma pharmacokinetics of fosfomycin and metronidazole after intraperitoneal administration in patients undergoing appendectomy for uncomplicated appendicitis. Fundam Clin Pharmacol. (2020). 10.1111/fcp.12535. [Epub ahead of print]. 31944378

[10] Moher D Hopewell S Schulz KF Montori V Gøtzsche PC Devereaux PJ. CONSORT 2010 Explanation and Elaboration: updated guidelines for reporting parallel group randomised trials. J Clin Epidemiol. (2010) 63:e1–37. 10.1016/j.jclinepi.2010.03.004 20346624

[11] Ioannidis JPA Evans SJW Gøtzsche PC O'Neill RT Altman DG Schulz K. Better reporting of harms in randomized trials: an extension of the CONSORT statement. Ann Intern Med. (2004) 141:781–8. 10.7326/0003-4819-141-10-200411160-00009 15545678

[12] Campbell MK Piaggio G Elbourne DR Altman DG. Consort 2010 statement: extension to cluster randomised trials. BMJ. (2012) 345:e5661. 10.1136/bmj.e5661 22951546

[13] Eldridge SM Chan CL Campbell MJ Bond CM Hopewell S Thabane L. CONSORT 2010 statement: extension to randomised pilot and feasibility trials. BMJ. (2016) 355:i5239. 10.1136/bmj.i5239 27777223 PMC5076380

[14] http://www.asahq.org/resources/clinical-information/asa-physical-status-classification-system (accessed March 4, 2020).

[15] Aldrete JA. Modifications to the postanesthesia score for use in ambulatory surgery. J Perianesth Nurs. (1998) 13:148–55. 10.1016/S1089-9472(98)80044-0 9801540

[16] Eypasch E Williams JI Wood-Dauphinee S Ure BM Schmülling C Neugebauer E. Gastrointestinal Quality of Life Index: development, validation and application of a new instrument. Br J Surg. (1995) 82:216–22. 10.1002/bjs.1800820229 7749697

[17] Eriksen JR Kristiansen VB Hjortsø N-C Rosenberg J Bisgaard T. [Effect of laparoscopic cholecystectomy on the quality of life of patients with uncomplicated socially disabling gallstone disease]. Ugeskr Laeger. (2005) 167:2654–6. 16014226

[18] Dindo D Demartines N Clavien P-A. Classification of surgical complications: a new proposal with evaluation in a cohort of 6336 patients and results of a survey. Ann Surg. (2004) 240:205–13. 10.1097/01.sla.0000133083.54934.ae 15273542 PMC1360123

[19] https://database.ich.org/sites/default/files/E6_R2_Addendum.pdf (accessed March 4, 2020).

[20] van Rossem CC Schreinemacher MHF Treskes K van Hogezand RM van Geloven AAW. Duration of antibiotic treatment after appendicectomy for acute complicated appendicitis. Br J Surg. (2014) 101:715–9. 10.1002/bjs.9481 24668341

[21] Van Rossem CC Schreinemacher MHF Van Geloven AAW Bemelman WA Van Acker GJD Akkermans B. Antibiotic duration after laparoscopic appendectomy for acute complicated appendicitis. JAMA Surg. (2016) 151:323–9. 10.1001/jamasurg.2015.4236 26580850

[22] Kleif J Rasmussen L Fonnes S Tibæk P Daoud A Lund H. Enteral antibiotics are non-inferior to intravenous antibiotics after complicated appendicitis in adults: a retrospective multicentre non-inferiority study. World J Surg. (2017) 41:2706–14. 10.1007/s00268-017-4076-6 28600695

[23] Fonnes S Holzknecht BJ Arpi M Rosenberg J. Intraperitoneal administration of fosfomycin, metronidazole, and granulocyte-macrophage colony-stimulating factor in patients undergoing appendectomy is safe: a phase II clinical trial. Sci Rep. (2019) 9:6727. 10.1038/s41598-019-43151-4 31040341 PMC6491470

[24] Grabein B Graninger W Rodríguez Baño J Dinh A Liesenfeld DB. Intravenous fosfomycin-back to the future. Systematic review and meta-analysis of the clinical literature. Clin Microbiol Infect. (2017) 23:363–72. 10.1016/j.cmi.2016.12.005 27956267

[25] http://mri.cts-mrp.eu/download/DE_H_1018_001_FinalSPC.pdf (accessed March 4, 2020).

[26] https://www.ema.europa.eu/documents/referral/tazocin-article-30-referral-annex-iii_en.pdf (accessed March 4, 2020).

[27] https://www.ema.europa.eu/documents/referral/zinacef-article-30-referral-annex-iii_en.pdf (accessed March 4, 2020).

