# Supplementary material for: Shorter Total Length of Stay After Intraperitoneal Fosfomycin, Metronidazole, and Molgramostim for Complicated Appendicitis: A Pivotal Quasi-Randomized Controlled Trial
Source: Front Surg. 2020 May 5;7:25. doi: 10.3389/fsurg.2020.00025 (PMC7214811; doi:10.3389/fsurg.2020.00025)
Supplement: Supplementary file 1 [file Data_Sheet_1.PDF]

Statistical analysis plan

for

Intraperitoneal administration of fosfomycin, metronidazole and molgramostim versus intravenous conventional antibiotics for perforated appendicitis – a pivotal quasi-randomized controlled trial

Agreement was reached on 21st January 2019 after discussion and prior to the statistical analyses.

The investigators of the study: Siv Fonnes, Jacob Rosenberg, Barbara Juliane Holzknecht, Magnus Arpi and Lars Nannestad took part in the discussion.

Table of content

Aim ... 3

Design ... 3

Outcomes ... 4

- Primary outcome ... 4
- Secondary outcomes ... 4
  - Questionnaires ... 4
  - Complications ... 4
  - Adverse events ... 6

Sample size ... 6

Baseline characteristics ... 7

Endpoints ... 7

- Primary endpoint ... 7
- Secondary endpoints ... 8
  - Questionnaires ... 8
  - Complications ... 9
  - Adverse events ... 10
- Missing data ... 10

Statistical methods ... 10

- Statistical program ... 11

Supplements ... 12

References ... 14

Aim

The aim of the trial was to investigate if intraoperative intraperitoneal administration of fosfomycin, metronidazole and recombinant human granulocyte-macrophage colony stimulating factor followed by oral antibiotic for three days (intervention group) was as effective as the current intravenous antibiotic treatment given during and three days (control group) after appendectomy for perforated appendicitis. We evaluated this through length of stay.

Design

Non-blinded, Q-RCT with a minimum of 6 participants in each group: intervention and control – a total of minimum 12 participants. The control and intervention group were included at two different hospitals.

We chose a quasi-randomized design to make the trial feasible within a manageable timeframe. The trial only included patients with appendix perforation. A rather large number of patients undergo surgery for suspected appendicitis, however, only around 75% of these patients are diagnosed with appendicitis at surgery [1]. Of these, only around 30% are diagnosed with complicated appendicitis at surgery [2]. So even if all possible patients were enrolled, only around 10-20% of the patients could be included in the trial. We anticipated that the feasibility of the trial would be increased with the design as a Q-RCT for three reasons. One, if each centre only had to implement one regimen for the seldom trial participant, it would be easier to ensure that this regimen could be applied day and night. Two, if the two groups of patients were included in parallel, the period of inclusion would be shorter because of the double catchment area for patient accrual. Three, the control group could be more efficiently included as only patients, where perforated appendicitis was found during laparoscopic appendectomy were approached and asked to participate.

We are convinced that the populations at Herlev Hospital and Bispebjerg Hospital, which are both located in the Copenhagen area, are comparable, so that the possible risk of bias was minimized. Both departments use the same surgical therapy for perforated appendicitis.

Outcomes

Primary outcome

Total length of in hospital stay (measured in hours) was defined as from end of the surgery until discharge from the hospital plus length of stay during a possible readmission within 30 days from surgery. The latter was calculated based on a review of the patient's record 30 days postoperatively (±3 days).

Secondary outcomes

Questionnaires

Gastrointestinal Quality of Life Index (GIQLI)

A disease-specific questionnaire [3] validated in Danish [4] was collected at 10 days postoperatively (±2 day) and at 30 days postoperatively (±3 days). The questionnaire evaluates the quality of life after surgery. It contains 36 questions, which explores five main domains: symptoms (19 items), physical status (seven items), emotions (five items), social functioning (four items), and the effect of medical treatment (one item). A five-point Likert-scale (0−4 points) is used to answer all items. The scale ranges from "never" to "all the time". A higher score denotes a better function.

Harms

A questionnaire regarding harms was filled out by the patients, see Supplements, Table 2. Questions about side effects was evaluated at the first, postoperative day and 10 days (±2 days) postoperatively when the patients had their sutures removed by the trial personnel. The questionnaire evaluates the possible harms of the trial treatment and was based on the answers of participants in of previous trial [5]. It contains seven questions, where the participant can describe if a possible harm has arisen. The scale has five possible answers, which ranges from "never" to "all the time". The final, eighth questions, gives the participant a possibility to describe any change they have experienced after the surgery.

Complications

Complication graded according to Clavien-Dindo

Postoperative complications will be graded according to the Clavien-Dindo grading [6]: information regarding complications is collected after surgery during hospital stay, 10 days

postoperatively (±2 day) when the patients had their sutures removed by the trial personnel and 30 days (±3 days) postoperatively through review of the patients' medical records and by the planned telephone interview. The definitions can be seen in Table 4.

Deep surgical site infections

Deep surgical site infection postoperatively: Information regarding surgical site infections was collected 10 days postoperatively (±2 day) when the patients had their sutures removed by the trial personnel and 30 days (±3 days) postoperatively through review of the patient's medical records and at the planned telephone call to the patients. It was defined as deep incisional surgical site infection according to Centre for Disease Control and Prevention (CDC) [7], which can be seen in Table 5.

Intraabdominal abscesses

Intraabdominal abscess: Information regarding intraabdominal abscesses was collected 10 days postoperatively (±2 day) when the patients had their sutures removed by the trial personnel and 30 days (±3 days) postoperatively through review of the patient's medical records and at the planned telephone interview. It was defined as an organ/space surgical site infection according to CDC [7] and can be seen in Supplements, Table 5. If a collection was found but not drained, it is not regarded as an abscess by definition in the current trial.

Readmissions

Readmissions: Information was collected 30 days (±3 days) postoperatively by review of the patient's medical records and at the planned telephone interview. Only readmissions related to the surgery was registered; e.g. admission and treatment of a non-related condition was not be registered.

Reoperations

Reoperations: Information will be collected 30 days (±3 days) postoperatively by review of the patient's medical records and at the planned telephone interview.

Convalescence

Time to return to normal activities

Time to return to normal activities: This time point was determined either 10 days (±2 days) postoperatively when the patients had their sutures removed by the trial personnel or 30

days (±3 days) postoperatively through the planned telephone interview of the participants. The date was defined at the time point at which the participant could return to normal daily activities.

Period of sick leave

Period of sick leave (absence from work): This time point was determined either 10 days (±2 days) postoperatively when the patients had their sutures removed by the trial personnel or 30 days (±3 days) postoperatively through the planned telephone interview of the participants. The parameter was defined as the number of days from the operation to the time point at where the participant returned to work or school.

Adverse events

Adverse events: registered by trial personnel during admission, 10 days (±2 days) postoperatively when the patients had their sutures removed by the trial personnel and 30 days (±3 days) postoperatively through review of the patient's medical records and at the planned telephone call to the patients.

Sample size

The power calculation for the trial was as follows. The cumulative 30-day length of stay was expected to be 72 hours with a standard deviation of 12 hours in the control group. When alfa was set at 0.05, beta was set at 0.80, and an acceptable difference between the groups was set at 48 hours, a randomised design requires 4 patients in each group, a total of 8 patients. However, as we expected data not to be normally distributed and planned to use a quasi-randomised design [8,9], we decided to include 6 patients in each group, a total of 12 patients to ensure sufficient power.

A participant has concluded the trial when the 30-day postoperative follow-up has been conducted. Inclusion of participants in the trial will continue until a minimum of six participants in each study group have reached the 30-day follow-up with data for the primary outcome parameter, total length of stay. Thus, we planned to include six patients in each study group and only if the primary outcome was not available at 30-day follow-up, further patients were included to ensure data on the primary outcome on six patients in each group.

Baseline characteristics

The baseline characteristics will be presented as depicted in Table 1. Age, height, weight, body mass index, number of times preoperative antimicrobial agents were administrated, and length of surgery will be presented as continuous numerical variables. Sex will be presented as a dichotomous variable, where the number of females and proportion in % are presented. American Society of Anesthesiologists (ASA) score will be presented as ordinal variable, where the number and proportion in % of each category (I, II, III, IV) is given. We expect that the data is not normally distributed due to the few number of participants. Because of the quasi-randomised design, a difference between groups could arise, therefore, the p-value of statistical tests will be reported. The rationale of presented variables and the statistical tests used is presented in Statistical methods.

Admission		Intervention group (n=x)	Control group (n=x)	p-value
Age, years		x (x-x)	x (x-x)	[from Mann-Whitney U-test]
Sex, female		n (%)	n (%)	[from Chi-square-test/Fisher's exact test]
Height, cm		x (x-x)	x (x-x)	[from Mann-Whitney U-test]
Weight, kg		x (x-x)	x (x-x)	[from Mann-Whitney U-test]
Body Mass Index, kg/m^2		x (x-x)	x (x-x)	[from Mann-Whitney U-test]
ASA score	I:	n (%)	n (%)	[from Chi-square-test /Fisher's exact test]
	II	n (%)	n (%)	
	II:	n (%)	n (%)	
	IV:	n (%)	n (%)	
**Preoperative antimicrobial agents***				
Times administrated		x (x-x)	x (x-x)	[from Mann-Whitney U-test]
**Surgery**				
Length, hours:minutes		xx:xx (xx:xx-xx:xx)	xx:xx (xx:xx-xx:xx)	[from Mann-Whitney U-test]

Table 1. Demographics of the xx included patients for continues variables in median (range) and for categorical variables in number (percent). ASA: American Society of Anesthesiologists [10]. ** Included the following antimicrobial agents: .*

Endpoints

Primary endpoint

The difference in total length of stay measured in hours will be compared between the intervention and the control group. Total length of stay was defined as from end of the operation until discharge from the hospital plus length of stay during a possible readmission

within 30 days from surgery. All randomised participants will be included in the analysis (intention-to-treat) if all data have been collected for 30 days postoperatively). The length of stay will be presented as a continuous numerical variable in the Result section of the manuscript. We expect that the data are not normally distributed due to the low number of participants, thus, medians and range for the intervention and control group will be presented. Furthermore, a Mann-Whitney U-test will be performed to compare the length of stay between the two groups.

Secondary endpoints

We expect that the data are not normally distributed due to the low number of participants. No statistical test will be used to compare the secondary endpoints in the two groups due to the lack of power. The results will therefore be descriptively presented.

Questionnaires

GIQLI

The total sum score and the score for each item of the questionnaire for the intervention and control group will be presented as continuous numerical variables for both the 10th and 30th postoperative day, as depicted in Table 2.

GIQLI	10th postoperative day		30th postoperative day	
	Intervention group (n=x)	Intervention group (n=x)	Intervention group (n=x)	Intervention group (n=x)
**Total**	x (x-x)	x (x-x)	x (x-x)	x (x-x)
Symptoms	x (x-x)	x (x-x)	x (x-x)	x (x-x)
Emotions	x (x-x)	x (x-x)	x (x-x)	x (x-x)
Physical function	x (x-x)	x (x-x)	x (x-x)	x (x-x)
Social function	x (x-x)	x (x-x)	x (x-x)	x (x-x)
Medical treatment	x (x-x)	x (x-x)	x (x-x)	x (x-x)

Table 2. The median (range) scores of the Gastrointestinal Quality of Life Index (GIQLI) [3] *overall and for each item of the intervention and the control group postoperatively.*

Harms

The answers from the questionnaire regarding harms will be reported descriptively for the two groups the intervention and the control group in the Results section of the manuscript. Harms that were present will reported as the number and proportion in % in each group.

Complications

The data on complications will be presented as a dichotomous variable, where the number of complications and proportion in % are presented for each group, as depicted in Table 3. Furthermore, deep surgical site infection, intraabdominal abscess, readmission and reoperations will be presented as dichotomous variables (with number and proportion in %) as depicted in Table 3 whereas Clavien-Dindo grading will be described in the text.

**Complications**	**Intervention group (n=x)**	**Control group (n=x)**
Overall complications	n (%)	n (%)
Deep surgical site infection	n (%)	n (%)
Intraabdominal abscess	n (%)	n (%)
Readmissions	n (%)	n (%)
Reoperations	n (%)	n (%)

Table 3. The number and proportion in per cent of complications in the intervention and the control group.

Convalescence

Time to return to normal activities

The time to return to normal activities in days will be presented both as dichotomous and continuous numerical variables in the Results section of the manuscript. The number and proportion in % of participants, who had returned within the 30th postoperative day, will be described. Dates for return will be used to calculate the continuous numerical variables. We expect that the data are not normally distributed due to the low number of participants, thus, medians and ranges for the intervention and control groups will be presented.

Period of sick leave

The period of sick leave in days will be presented both as dichotomous and continuous numerical variables in the Results section of the manuscript. The number and proportion in % of participants, who were still on sick leave at the 30th postoperative day, will be described. Dates for return from sick leave will be used to calculate the continuous numerical variables.

We expect that the data are not normally distributed due to the low number of participants, thus, medians and ranges for the intervention and control groups will be presented.

Adverse events

Adverse events and serious adverse events will be presented as dichotomous variables, where the number of complications and proportion in % are presented for each group in the Results section of the manuscript, furthermore, details on the adverse events will be will described.

Missing data

Missing data regarding the primary outcome, length of stay, will be excluded from the analysis as total length of stay was defined from the end of surgery until 30 days postoperatively.

Missing data in the secondary outcomes regarding the questionnaires (GIQLI and harms) will be treated as follows. All received GIQLI questionnaires regardless of the amount of missing data will be included. If the questionnaire was never received data will be excluded. All received questionnaires regarding harms will be reported regardless of missing data.

Missing data of the secondary outcomes complication and adverse events due to lack of 30th day contact with participant cannot be reported. If no dates have been registered for the secondary outcomes of convalescence the data are excluded from the calculations of the continuous numerical variables.

Statistical methods

Continuous numerical data are reported as mean and standard deviation if normally distributed. If not normally distributed, continuous numerical data are reported as median and range. We test for normality by visual inspection of histograms and Q-Q plots. Normally distributed, independent, continuous data are analysed with the parametric t-test. Not normally distributed, independent, continuous data are analysed with the non-parametric Mann-Whitney U-test (Wilcoxon sum-rank test).

Binary, categorical data are reported as number and proportion in %. These will be compared between the intervention group and the control group with Chi-square-test, or if any of the expected cell counts are <5, the p-value from Fisher's exact test is reported.

A p-value ≤0.05 is considered statistically significant.

Statistical program

Data will be analysed using SAS Enterprise Guide 7.1 (SAS Institute Inc., USA).

Supplements

Grade	Definition
Grade I	Any deviation from the normal postoperative course without the need for pharmacological treatment or surgical, endoscopic, and radiological interventions<br>Allowed therapeutic regimes are: drugs as antiemetics, antipyretics, analgetics, diuretics, electrolytes, and physiotherapy. This grade also includes wound infections opened at bedside.
Grade II	Requiring pharmacological treatment with drugs other than such allowed for grade I complications<br>Blood transfusions and total parenteral nutrition are also included
Grade III	Requiring surgical, endoscopic or radiological intervention
Grade IIIa	Intervention not under general anesthesia
Grade IIIb	Intervention under general anesthesia
Grade IV	Life-threatening complication (including CNS complications)* requiring IC/ICU management
Grade IVa	Single organ dysfunction (including dialysis)
Grade IVb	Multiorgan dysfunction
Grade V	Death of a patient
Suffix “d”	If the patient suffers from a complication at the time of discharge, the suffix “d” <(for “disability”) is added to the respective grade of complication. This label indicated the need for a follow-up to fully evaluate the complication

Table 4. Clavien-Dindo classification of surgical complications.[6] **Brain hemorrhage, ischemic stroke, subarrachnoidal bleeding, but excluding transient ischemic attacks. CNS, central nervous system; IC, intermediate care; ICU, intensive care unit.*

Deep incisional surgical site infection

Infection occurs within 30 days after the operation if no implant† is left in place or within 1 year if implant is in place and the infection appears to be related to the operation

And

infection involves deep soft tissues (e.g., fascial and muscle layers) of the incision

and at least *one* of the following:

1. Purulent drainage from the deep incision but not from the organ/space component of the surgical site.
2. A deep incision spontaneously dehisces or is deliberately opened by a surgeon when the patient has at least one of the following signs or symptoms: fever (>38ºC), localized pain, or tenderness, unless site is culture-negative.
3. An abscess or other evidence of infection involving the deep incision is found on direct examination, during reoperation, or by histopathologic or radiologic examination.
4. Diagnosis of a deep incisional surgical site infection by a surgeon or attending physician.

Notes:

1. Report infection that involves both superficial and deep incision sites as deep incisional surgical site infection.
2. Report an organ/space surgical site infection that drains through the incision as a deep incisional surgical site infection.

Organ/space surgical site infection

Infection occurs within 30 days after the operation if no implant† is left in place or within 1 year if implant is in place and the infection appears to be related to the operation

and

infection involves any part of the anatomy (e.g., organs or spaces), other than the incision, which was opened or manipulated during an operation

and at least *one* of the following:

1. Purulent drainage from a drain that is placed through a stab wound‡ into the organ/space.
2. Organisms isolated from an aseptically obtained culture of fluid or tissue in the organ/space.
3. An abscess or other evidence of infection involving the organ/space that is found on direct examination, during reoperation, or by histopathologic or radiologic examination.
4. Diagnosis of an organ/space surgical site infection by a surgeon or attending physician.

Table 5. CDC's criteria for defining a surgical site infection. [7] *† National Nosocomial Infection Surveillance definition: a nonhuman-derived implantable foreign body (e.g., prosthetic heart valve, nonhuman vascular graft, mechanical heart, or hip prosthesis) that is permanently placed in a patient during surgery. ‡ If the area around a stab wound becomes infected, it is not an SSI. It is considered a skin or soft tissue infection, depending on its depth.*

References

[1] Jørgensen AB, Amirian I, Watt SK, Boel T, Gögenur I. No circadian variation in surgeons' ability to diagnose acute appendicitis. *J Surg Educ* 2016;73:275–80.

[2] Kleif J, Rasmussen L, Fonnes S, Tibæk P, Daoud A, Lund H, et al. Enteral antibiotics are non-inferior to intravenous antibiotics after complicated appendicitis in adults: a retrospective multicentre non-inferiority study. *World J Surg* 2017;41:2706-14.

[3] Eypasch E, Williams JI, Wood-Dauphinee S, Ure BM, Schmülling C, Neugebauer E, et al. Gastrointestinal Quality of Life Index: development, validation and application of a new instrument. *Br J Surg* 1995;82:216–22.

[4] Eriksen JR, Gogenur I, Rosenberg J. [Laparoscopic surgery for incisional hernia]. *Ugeskr Laeger* 2007;169:3557.

[5] Fonnes S, Holzknecht BJ, Magnus Arpi M, Jacob Rosenberg J. The safety of intraperitoneal administration of fosfomycin, metronidazole, and granulocyte-macrophage colony-stimulating factor in patients undergoing appendectomy for appendicitis: a phase II clinical trial (submitted).

[6] Dindo D, Demartines N, Clavien P-A. Classification of surgical complications: a new proposal with evaluation in a cohort of 6336 patients and results of a survey. *Ann Surg* 2004;240:205–13.

[7] Mangram AJ, Horan TC, Pearson ML, Silver LC, Jarvis WR. Guideline for prevention of surgical site infection, 1999. Hospital Infection Control Practices Advisory Committee. *Infect Control Hosp Epidemiol* 1999;20:250-78.

[8] Kerry SM, Bland JM. Sample size in cluster randomisation. *BMJ* 1998;316:549.

[9] Kerry SM, Bland JM. Analysis of a trial randomised in clusters. *BMJ* 1998;316:54.

[10] http://www.asahq.org/resources/clinical-information/asa-physical-status-classification-system (last accessed January 21, 2019).
